# Association of 7-Day Profiles of Motor Activity in Marital Dyads with One Component Affected by Parkinson’s Disease

**DOI:** 10.3390/s23031087

**Published:** 2023-01-17

**Authors:** Marco Rabuffetti, Ennio De Giovannini, Ilaria Carpinella, Tiziana Lencioni, Luca Fornia, Maurizio Ferrarin

**Affiliations:** 1 IRCCS Fondazione Don Carlo Gnocchi, 20148 Milano, Italy; 2 Centro Medico Riabilita Cooperativa Sociale Mano Amica Onlus, 36015 Schio, Italy; 3 Department of Medical Biotechnology and Translational Medicine, Università degli Studi di Milano, 20133 Milano, Italy

**Keywords:** social actigraphy, Parkinson’s disease, dyad, cross-correlation, motor activity, rehabilitation

## Abstract

(1) Background: A noticeable association between the motor activity (MA) profiles of persons living together has been found in previous studies. Social actigraphy methods have shown that this association, in marital dyads composed of healthy individuals, is greater than that of a single person compared to itself. This study aims at verifying the association of MA profiles in dyads where one component is affected by Parkinson’s disease (PD). (2) Methods: Using a wearable sensor-based social actigraphy approach, we continuously monitored, for 7 days, the activities of 27 marital dyads including one component with PD. (3) Results: The association of motor activity profiles within a marital dyad (cross-correlation coefficient 0.344) is comparable to the association of any participant with themselves (0.325). However, when considering the disease severity quantified by the UPDRS III score, it turns out that the less severe the symptoms, the more associated are the MA profiles. (4) Conclusions: Our findings suggest that PD treatment could be improved by leveraging the MA of the healthy spouse, thus promoting lifestyles also beneficial for the component affected by PD. The actigraphy approach provided valuable information on habitual functions and motor fluctuations, and could be useful in investigating the response to treatment.

## 1. Introduction

Parkinson’s disease (PD) is a chronic neurodegenerative disease affecting approximately 7 million people worldwide [1]. PD progressively affects motor capacity and cognitive functions [2], leading to reduced quality of life [2], loss of independence [3], and, consequently, high costs in terms of burden on caregivers, as well as on national health and social services [4].

Freezing of gait [5], festination [6], gait initiation [7] and termination deficits [8], poor dynamic balance [9,10], difficulty in negotiating turning [11], tremor [12], and upper limb locomotor synergies [13] are among the typical motor signs affecting persons with PD. More generally, PD impacts many aspects of daily life, including sleep and diet [14]. Generally, the motor symptoms related to PD can be studied with the proper laboratory setup, namely in movement analysis laboratories [15,16,17]. However, more and more often, wearable sensors [18,19,20,21,22,23] and related actigraphic methods have been considered [24,25,26], especially for the possibility of studying patients’ behaviour in their everyday life [27], thus quantifying performance and capacity [28] in an ecological environment and complementing the laboratory analysis.

A relevant aspect of any chronic and progressive disease, and of PD in particular, is the effect on the social network of the person, namely their co-living relatives, and particularly their spouses, the other component of the marital dyad [29,30,31]. Specifically, physical activities are among the domains of a marital dyad on which a chronic disease may have a great influence: there is evidence of a concordance in sedentary behaviours and physical activities between two spouses, both in a healthy couple and a couple where one component is affected by a disease [32,33,34].

### 1.1. Related Works

Few authors have focused on the modulation of motor behaviour by social interactions, despite several reasonable observations which would support such a modulation: humans often move together, complying not only to social constraints (school and work timetables, shops’ openings, sleep timing etc.), but also to implicit hints from those living close [35,36]. Coherent with these phenomenological observations, in the last decades several neuroscientific studies have shown the presence within our central nervous system of parietal–frontal circuits encoding actions performed by others, allowing a first-person grasp of the motor goals and intentions of other individuals [37]. Thanks to this mechanism, the observed other represents a rich source of social affordances for the self [38]. Interestingly, it has been shown experimentally that concurrent upper limb movements performed by two subjects face to face show a coupling effect [39], and some authors have proposed that humans tend to adopt similar behaviours in everyday life [40]. This association has been observed in parent–son dyads [41], and has been considered able to facilitate physical activity [42]. Questionnaire approaches have been used to detect such association in everyday motor activities [43]. Recently, some authors have faced the problem of detecting association in motor activities by using actigraphic methodology based on wearable sensors [44,45,46]. Harada [44] objectively quantified, with an accelerometer, the daily sedentary behaviours, for seven days, of 72 marital dyad components, and found a fairly good association between the couples’ components. Ashe [45] enlarged the previous assessment to 112 familiar dyad components, confirming the good association. Pauly stressed the aspect of synchronicity by assessing intra-couple covariations of 7-day accelerometric profiles of motor activity on an hourly basis [46]. Their results, from 414 couples pooled from two different studies, showed a Synchrony of Moderate to Vigorous Physical Activity (MVPA) with cross-correlation coefficients ranging from 0.35 to 0.42, and a Synchrony in Sedentary Activity which ranged from 0.36 to 0.39.

### 1.2. Contributions

The authors of the present study proposed an actigraphic methodology, termed social actigraphy [47], based on wearable accelerometers, to quantify the association between the motor activity profiles of the two components of a dyad within 1-min epochs, which is short enough to describe such an association in terms of movement simultaneity. This method was applied to married couples of healthy elderly persons. In particular, the data from 20 married couples demonstrated that the association between the profiles of motor activity of the two partners belonging to the same couple (correlation coefficient 0.444) was significantly higher than the association between the profiles of individual motor activity on different days (correlation coefficient 0.335) [47]. Such strong interpersonal association is surely promoted by a common lifestyle and preferences. Obviously, the pathological condition of one of the couple’s partners was expected to interfere with such an association.

### 1.3. Aims

The aims of the present study, to be fulfilled by an experimental study with cross-sectional observational design, are

- to quantify the association between the motor activity levels of two components of a marital dyad living together, with one component being affected by Parkinson’s disease (aim 1);
- to analyse the interplay between disease severity (as quantified by the UPDRS III score) and the symmetry between magnitudes of motor activity levels, as previously identified (aim 2).

There is no a priori expected trend of the association between the spouses’ motor activities (aim 1). In fact, the disease affecting one component of the dyad might be correlated with either increased association, in case of motivation to maintain good physical fitness, or with reduced association, if the severity of the disease prevents the affected partner from engaging in physical activity similar to that of the healthy partner.

As to the second aim, it is expected that the healthy component within a couple has a higher motor activity compared to the partner affected by Parkinson’s disease. Moreover, it is hypothesised that a greater severity of the disease is associated to a larger asymmetry between the motor activity of the two components and to a smaller association between the motor activity levels of the marital dyads.

## 2. Materials and Methods

This is a non-blind, observational cross-sectional study comparing different actigraphic variables recorded from a population of marital dyads with one component affected by Parkinson’s disease.

### 2.1. Participants’ Enrollment

Participants were identified from a larger cohort of individuals who underwent actigraphic recordings, routinely administered at the Mano Amica Onlus Social Rehabilitation Centre (Schio, Vicenza, Italy), to support the clinical assessment of people with Parkinson’s disease. The partners of the patients were possibly enrolled to provide normative actigraphic data.

The inclusion criteria were: to be 50 years old or above, to belong to a cohabiting couple whose components are a person with Parkinson’s disease and a healthy partner, to be able to walk unassisted (Hoehn and Yahr scale ≤4), and to have concurrent actigraphic recordings of both components of the couple.

The exclusion criteria were: coxarthrosis, gonarthrosis, dementia, dysphagia, and dyskinesia.

All assessments were managed by a multidisciplinary team from the recruitment site institution, including a neurologist, a physiatrist, a physical rehabilitation therapist, a logopedist, and a neuropsychologist.

Patient enrolment and data collection were performed within the frame of the clinical activity of co-author E.D.G., according to the ethical standards of the responsible committee on human experimentation (institutional and national) and the Helsinki Declaration of 1975, revised in 2000. All participants provided a signed informed consent to participate in the study and to let their data to be analysed and presented in anonymous format for research aims.

### 2.2. Data Collection and Analysis

All subjects wore, for 7 consecutive days, a wearable actigraph (Geneactiv, Activinsights, Huntingdon, UK) on their non-dominant wrist, without removing it except for discomfort or health-related issues. The device was waterproof, and therefore compatible with water- and hygiene-related activities. Each participant with PD underwent a clinical visit, during which an expert clinician rated the severity of motor symptoms using the MDS-Unified Parkinson Disease Rating Scale (MDS-UPDRS) part III (range between 0 (no impairment) and 108 (severe impairment)). Data recording began following the clinical visit and was synchronized between the two individuals of each dyad.

The wrist-worn tri-axial sensor measured, uninterruptedly for 7 days, acceleration at a sampling rate of 100 Hz and stored the data on an on-board memory resident. These raw data were analysed according to the method detailed in [48,49], and are briefly reported below.

**Figure 1:**
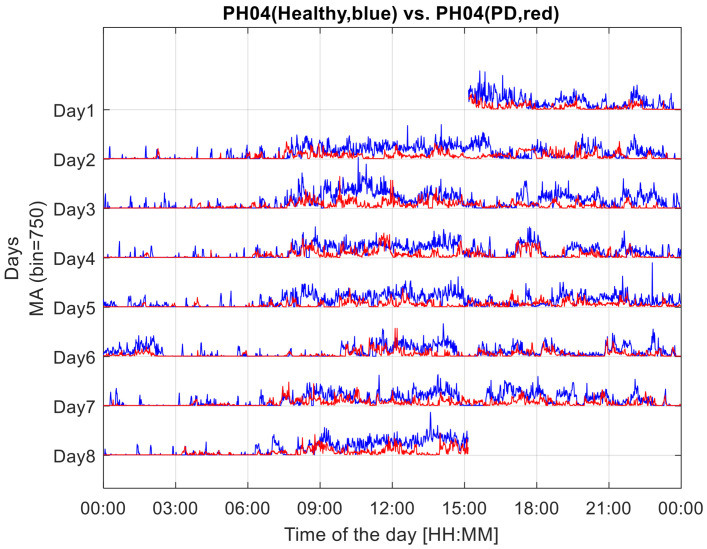
Seven-day profiles of MA, Motor Activity indices, computed in 1-min epochs from the recordings of the two components of a couple, the experimental dyad. The MA profile of the healthy component of the dyad is drawn in blue. That of the dyad component with Parkinson’s disease is drawn in red.

The method’s first step consists of the segmentation of the profile of the acceleration vector norm in epochs of 1 min, thus identifying 1440 epochs per day and 10,080 epochs in the 7-day overall duration of the monitoring. For each epoch, a single value was computed as the standard deviation of the acceleration vector norm (composed of 6000 values). This index, termed the Motor Activity (MA) index, was intended to quantify the 7-day profile of the level of motor activity, as assessed at the sensor level (see Figure 1). MA is an index that quantifies global motor activity, as it is calculated from data of functional movements, including locomotor functions, which imply an acceleration pattern characterising the wrist.

The second step consists of computing an index that quantifies the association between the two profiles of the MA index. Originally, the adopted method was intended to consider intra-subject MA profiles from the right and left wrist sensors, while, in the transposition at the social level, the social actigraphy approach presented here intends to apply the same metrics and algorithms to inter-subject MA profiles from two individuals. The applied model implies the quantification of the correlation coefficient determined by the two profiles. The implementation is based on the “corrcoef” operator in the MATLAB computing environment (R2016a, The Mathworks, Natick, MA, USA). The resulting correlation coefficient (CC), the primary outcome, quantifies the association between the two profiles, i.e., how much the shape of the two profiles follows a common pattern. It is relevant to note that the different magnitudes of the two profiles are not determinant for the computation of the CC index. The index ranges from the value 1, meaning that the two MA patterns have the same shape but not necessarily the same amplitude, down to the null value 0, meaning an absence of any correlation. Analogously, negative values from −1 to 0 denote a relation of one profile to the other, with the sign changed.

**Figure 2:**
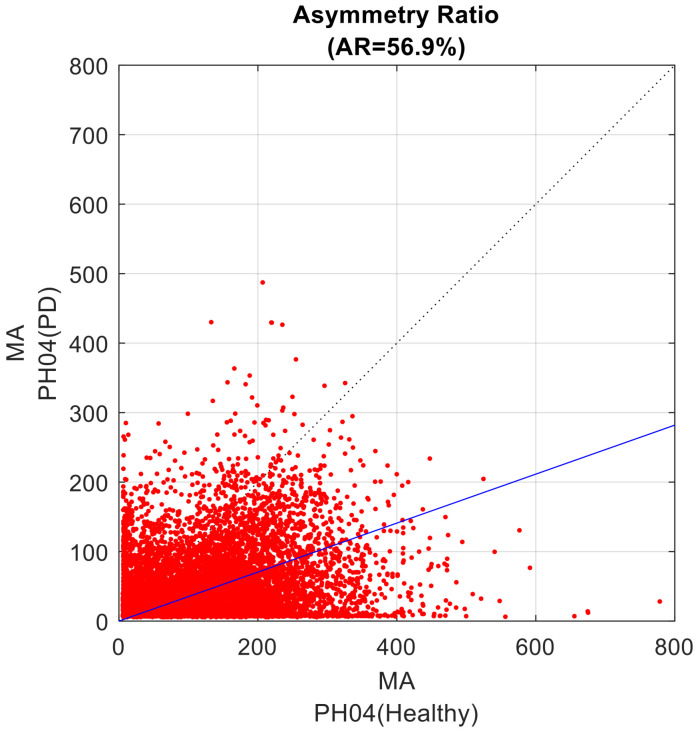
Scatter plot of the two considered MA profiles. The values from the healthy component of the dyad are set to the X-axis; the values of the dyad component with PD are set to the Y-axis. The data cloud best fitting line is presented in blue; the bisect line is the dotted line. The AR value of the present example is 56.9%.

The third step is based on the analysis of the relative amplitude of the common-mode pattern between the two considered MA profiles. From the scatter plot of one MA profile vs. the other MA profile (Figure 2), the data cloud best-fitting line was identified as the line passing through the axes’ origin and minimizing the sum of squared residuals [50]. The resulting line is the geometrical entity that summarizes the symmetry that occurred in the recordings. This line computationally corresponds to the first eigenvector, i.e., the common mode, as obtained by a singular value decomposition in a principal component analysis.

The inclination angle α, ranging from 0° to 90°, of the best fitting line relative the abscissa axis, supports the computation of the Asymmetry Ratio (AR) index, a secondary outcome, according the following formula:

AR = 100.0 × (45° − α)/45°

Such definition provides an AR index able to quantify how unbalanced the contribution is of the two original profiles to the first eigenvector: a 0% value is for perfectly balanced weights of the two MA profiles in defining the first component (and the fitting line coincides with the quadrant bisect line), progressively increasing up to 100%, which denotes progressively increasing weight of the first MA profile; conversely, a progression from 0% to −100% evidences that the second MA profile is preponderant.

The code involved in the analysis was developed by the authors in the MATLAB environment, release R2016a (The Mathworks, Natick, MA, USA), and is available to any interested reader upon direct request to the corresponding author.

### 2.3. Statistical Analysis

The overall dataset consisted of 54 individuals, i.e., 54 MA profiles, matched two by two according to the dyads.

By directly applying the method proposed in [47], to all possible combinations of two MA profiles, either from the components of a dyad (intra-dyad) or between unrelated individuals (extra-dyad), the following indices were obtained:

- CC_couple_PH—intra-dyad CC quantifying the correlation between the activity profiles of a person with PD and a healthy individual belonging to the same dyad, 27 values;
- CC_between_HH—extra-dyad CC quantifying the correlation between the activity profiles of two healthy individuals belonging to different dyads, 351 values;
- CC_between_PP—extra-dyad CC quantifying the correlation between the activity profiles of two individuals with PD belonging to different dyads, 351 values.
- Furthermore, when comparing any single MA profile with itself being shifted by 24 h and 12 h respectively, the following indices were computed:
- CC_self24_H—intra-personal CC (comparing an individual MA profile with the 24 h-shifted self, computed from the 6-day overlapped profile sections) quantifying an intra-individual day-to-day variability in healthy individuals, 27 values;
- CC_self24_P—intra-personal CC (comparing an individual MA profile with the 24 h-shifted self, computed from the 6-day overlapped profile sections) quantifying an intra-individual day-to-day variability in individuals with PD, 27 values;
- CC_self12_H—unrelated intrapersonal CC (comparing an individual MA profile with the 12H-shifted self, computed from the 6.5-day overlapped profile sections) quantifying an intra-individual day-to-night variability in healthy individuals, 27 values;
- CC_self12_P—unrelated intrapersonal CC (comparing an individual MA profile with the 12H-shifted self, computed from the 6.5-day overlapped profile sections) quantifying an intra-individual day-to-night variability in individuals with PD, 27 values.
- The additional Asymmetry Ratio (AR) index, quantifying the unbalance between two MA profiles, was computed for the intra-dyad condition only:
- AR_couple_PH—asymmetry ratio between two MA profiles, one from a person with PD and one from a healthy individual, belonging to the same dyad, 27 values.

The association between indices and UPDRS III was investigated through a linear interpolation analysis.

The resulting CC table was analysed by non-parametric tests given the small sample size: an analysis of variance (Kruskal–Wallis), followed by post-hoc multiple comparisons (Wilcoxon–Mann–Whitney, as implemented by the “ranksum” MATLAB function). *p*-value was set to 0.05 and was corrected according to the Bonferroni method for multiple comparisons. All analyses were implemented by the authors in MATLAB R2016a (The Mathworks, Natick, MA, USA).

## 3. Results

Fifty-four individuals (27 persons with PD, 19 males and 8 females, aged 61–87 years, mean 71 years, and 27 healthy partners, 8 males and 19 females, aged 57–81 years, mean 69 years) from 27 married cohabitant couples (the considered dyads) were enrolled. All participants were already retired from their jobs, were Italian, and were living in the area north of Vicenza (Italy). All participants with PD followed an individually tailored drug therapy based on dopamine-mimetic drugs and/or L-dopa. One patient assumed drug therapy by a duodenal pump. One patient had a deep-brain-stimulation surgical implant. Five patients intermittently used a walking aid (two used a cane, three used a walker). No specific indication for programs involving motor activities was given to the participants, since participants with PD were already involved in one or two weekly group sessions of physical therapy programmes, cognitive rehabilitation, and logotherapy.

**Figure 3:**
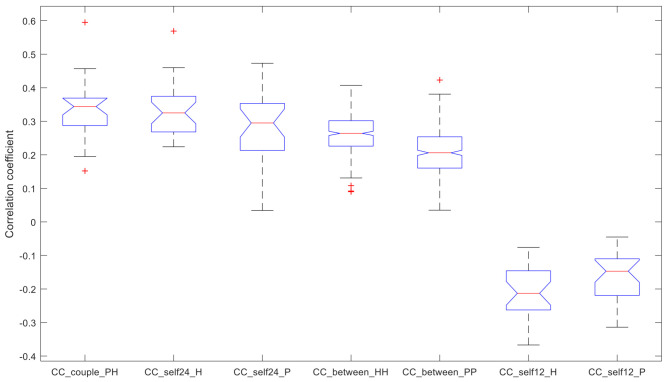
Correlation Coefficient (CC) of the identified analyses. Index CC quantifies the association between two MA profiles (+1 for perfect association, 0 for no association, −1 for perfect opposite association). Each box plot reports median, quartiles, max and min values; possible outliers are marked with a “+”.

A summary of the resulting CC index values is reported in the boxplot in Figure 3.

The post-hoc analysis (significant *p*-value after Bonferroni correction *p* = 0.05/21 = 0.0024) produced the following Table 1 of *p*-values (shown in bold characters where significant).

Therefore, the statistical tests showed that:

- the within-couple correlation CC_couple_PH (median value 0.344), the within-healthy correlation CC_self24_H (0.325), and the within-PD correlation CC_self24_P (0.295) are comparable;
- CC_couple_PH and CC_self24_H have higher values compared to the remaining indices;
- CC_self24_P has higher values compared to the remaining indices, except for the between-healthy correlation CC_between_HH (0.264);
- CC_between_HH has higher values compared to the between-PD correlation CC_between_PP (0.206);
- CC_between_HH and CC_between_PP have higher values than CC_self12_H (−0.213) and CC_self12_P (−0.147);
- CC_self12_H and CC_self12_P have no difference between them.

**Figure 4:**
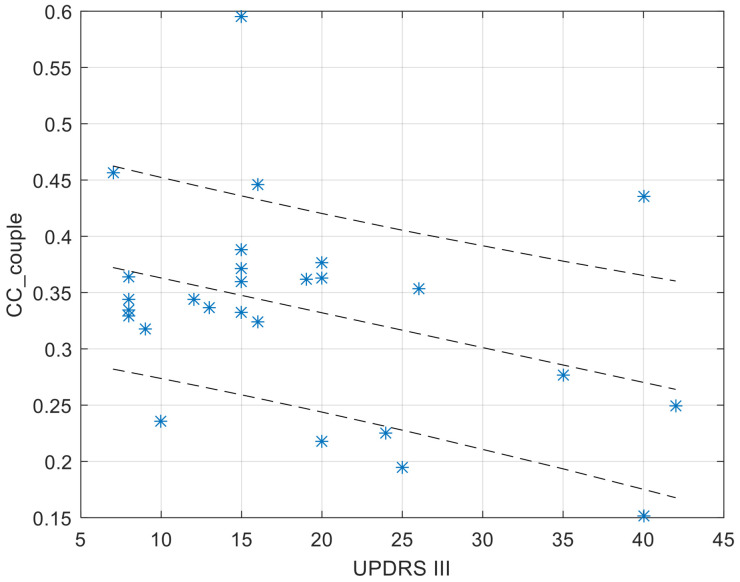
Values of CC_couple_PH in function of the UPDRS score of the dyad component with PD. The regression function is completed by its 95% confidence interval.

**Figure 5:**
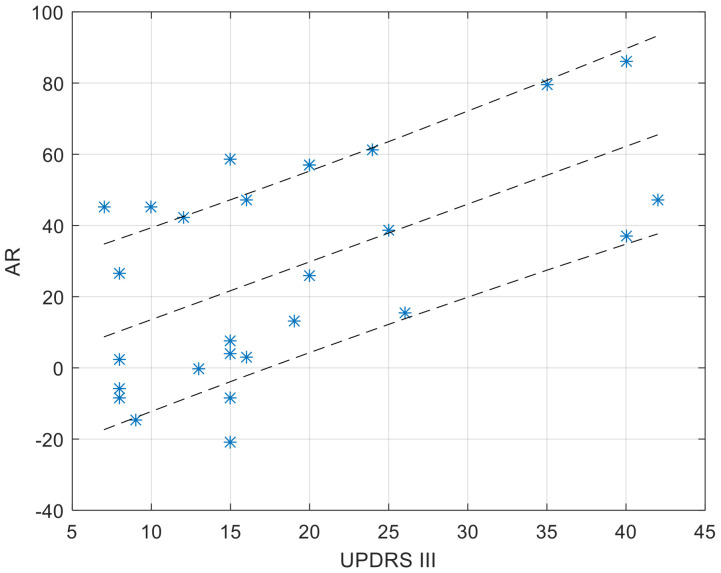
Values of AR_couple_PH as a function of the UPDRS score of the dyad component with PD. The regression function is completed by its 95% confidence interval. A positive AR value (up to a max of 100%) denotes larger values in the MA profile of the healthy dyad component.

The CC (Figure 4) and AR (Figure 5) values of the within-couple correlation “CC_couple_PH” group are presented along with the degree of impairment of the dyad component with PD, as quantified by the UPDRS III score. The scatterplots reported below were completed by the linear regression interpolation line, with 95% confidence intervals. It is apparent that the within-couple correlation CC_couple_PH tends to decrease with the increase of UPDRS III (i.e., greater clinical impairment), and that the within-couple asymmetry AR tends to increase with the increase of UPDRS III.

## 4. Discussion

The monitoring procedures were performed without problems and the wrist-worn sensor was well accepted by participants; no issues were reported. This is of particular importance since there is a lack of longitudinal studies focused on the motor activity of persons with PD, as was recently pointed out concerning gait [51]. In addition, the identification of low-cost sensing technology, such as the one presented here, able to quantitatively assess motor activity during daily living activities in the home and community settings, is a clinical need.

The design of the present data collection has some aspects which are replications of what was already reported in the 2022 article: some of the CC coefficient indices are fully comparable with some of the results presented in [47]: CC_self24H (0.325 vs. 0.335, respectively, −3%), CC_between_HH (0.264 vs. 0.277, −5%), and CC_self12H (−0.213 vs. −0.203, +5%). Such comparable figures ensure that the present data set is consistent and can be compared to the already published data set [47] without biases or distortions.

In the present data set, we can detect a major aspect which differentiates it from the data set of healthy marital dyads [47]: the CC_couple_PH (the within-couple association between a healthy component and a component with PD) is not significantly larger than the CC_self24H and CC_self24P (the between-day associations of healthy and PD dyad components, respectively), as was found in the previous data set from healthy dyads.

The remaining comparisons are substantial replicas of what has already been observed: the correlation within individuals (CC_self24) is slightly larger than the correlation between individuals (CC_between), and the association is completely disrupted when comparing an individual to its profile 12 h apart (CC_self12). The previous statements are true for both healthy participants and those with PD.

The major evidence implies that, when considering the whole data set composed of marital dyads with one component affected by PD, the correlation between the two motor activity profiles is comparable with what was observed within each participant (the association between subsequent days’ motor profiles) and between any pair of unrelated individuals. This result is different from what was found in couples composed of both healthy individuals [47], where the motor activity correlation of the couple benefited from an increase compared to the activity of a single individual. A straight forward interpretation might lead to the conclusion that the PD symptoms affecting a component of a marital couple might disrupt the increase in behavioural association that characterises the dyad itself. However, since PD is a progressive degenerative disease, such conclusion must necessarily be analysed in relation to the severity of the disease. A perusal of the values of the intra-dyad cross-correlation (CC_couple_PH) vs. the clinical UPDRS III score, which quantifies the severity of PD motor symptoms, showed that the more severe the symptoms, the lower the CC_couple_PH values. Accordingly, we must restate our previous conclusion. Early-stage PD does not disrupt the association between the motor profiles of two marital dyad components, but this disruption progressively occurs with the progression of the disease’s symptoms. Interestingly, it has been shown that persons with PD have a decreased response in the basal ganglia and the mirror system, hypothesising that this reduced activation may be related to a disruption of the cognitive resonance mechanisms, thus impairing the perception of others’ actions [52].

This aspect is relevant to the role that the marital dyad can play in moulding the motor profile: in an early stage of the disease, there is a strong association between the two dyad components which can be exploited to attract the person with PD towards a more active lifestyle, if the healthy partner himself shows an active lifestyle, with consequent beneficial aspects for his/her condition. With the progression of the disease’s symptoms, such associations are slightly reduced, but do not disappear.

As to the comparison of the activity level, to identify who is more or less active within the couple, an Asymmetry Ratio index was computed to compare motor profiles. The results reported here are related to associations within the marital dyad (AR_couple). A perusal of data showed that AR_couple is directly correlated to progression of PD symptom severity: the higher the UPDRS III score, the larger the AR_couple value, with a prevalence towards the healthy partner. This fully confirms the preliminary hypothesis (aim 2 of the study); namely, that the healthy component of the couple is prevalent and may induce an imbalance between MA profiles.

It is interesting to note that, despite the fact that motor profile association (CC value) is not mathematically related to motor activity asymmetry (AR value), a more severe PD condition implies lower CC and higher AR values, while, conversely, a less severe condition implies higher CC and lower AR. This depicts a situation in which couples where the component with PD is at an early stage of the disease are quite synchronized, and show a milder prevalence, if at all, of the healthy partner concerning the intensity of physical activities. Conversely, at an advanced stage of the disease, the two components of a marital dyad with one component with PD tend to disconnect the respective profiles of motor activity, while the unbalance of the intensity of motor activities becomes larger.

The evidence that disease progression tends to disrupt the couple unity of motor behaviour in their daily performances is compatible with the observation that this involves motor capacity [53], and, in general, many functional domains [31].

Regarding the utility of these results in terms of clinical management of a person with PD, it is apparent that, at an early stage of the disease, the high level of association between the motor profiles within couples appears to be a potential tool to increase the activity level of the individual with PD, with consequent beneficial outcomes. In fact, though an association does not imply a causal relation, a healthy partner leading an active lifestyle could result in a more active lifestyle of the partner with PD, and in general in a higher relationship satisfaction at the emotional level [54,55]. Not forgetting that such a goal, i.e., having a more active lifestyle together, is also beneficial for the healthy partner and may help to contrast the difficulties that might show up in life [55]. As to the aim of the study, the present results suggest that living together does not produce the same figure in mixed healthy–PD couples compared to couples composed of two healthy partners. Still, it is likely that this is due to the inclusion of persons severely affected by PD. When focusing on less severely affected persons, the CC tends to increase and asymmetry tends to decrease, according to what was observed in healthy subjects.

As a group, the couples with one component affected by PD showed a level of association between motor profiles which was comparable to the association observed between different days of the same subject. However, when adopting this method to classify the status of each couple, it is possible to identify couples whose between-partner association of motor profiles is more pronounced.

Given the fundamental role played by physical activity in the management of patients with Parkinson’s disease, any strategy oriented at promoting a more active lifestyle is relevant [51]. The presented evidence suggests that, particularly in early phases of Parkinson’s disease, the relationship of the patient with active family members might contribute to promoting an active lifestyle. This can take place along with new approaches made available by modern technologies: the IoT (Internet of Things) paradigm involves having sensors placed in all environments, and possibly supporting the application of Artificial Intelligence algorithms to promote an active lifestyle of the persons living in those environments, whether they are private or public ones [56]. Moreover, another possible way to promote an active lifestyle is to use virtual reality platforms, entering the houses of a relevant part of the population these days, to implement specific exergames oriented towards the management of patients with neurodegenerative disorders, including PD [57]. Preliminary results have shown that the adoption of ambient and body sensors is accepted and favoured by patients in the management of their condition [58].

### Limitations

Due to the particular couple-matched observational nature of the present study, no control group was included, though our previous study conducted on healthy elderly people provided reference control data (Rabuffetti et al. [47]).

Data collection took place between April 2019 and June 2021, with exclusion of the phase of strict lockdown due to the COVID-19 pandemic (18 couples before the pandemic, seven couples in the summer of 2020 when restrictions due to the pandemic had been lifted, and two in 2021 after the introduction of COVID-19 vaccines). The exclusion of the strict lockdown phase is expected to minimize possible confounding effects due to the pandemic.

## 5. Conclusions

In conclusion, the hypothesis that the healthy component in a mixed couple might “pull” the partner with PD (in terms of increasing the association between the two motor activity profiles) is more likely to happen when the disease is not severe. Moreover, rehabilitation operators (therapists, psychologists, and physicians) might assume the role of educators of the healthy partner of the person with PD [59]. It will not be simply a matter of asking the healthy partner to lead a more active life, but of educating the healthy partner in creating occasions for moving together. It is relevant to note that moving together is expected to stimulate mental functions involving observation and mirroring [39], and this is expected to involve a benefit for the patient with PD. In particular, the vicarious observation of actions performed by others has been shown to have a profound impact on the sensorimotor system of the observer, not only in terms of action perception, but also in terms of motor pathways excitability. This mechanism has been shown to have several implications, ranging from acquisition of motor skills [60] to action understanding and social cognition [37]. Moreover, this mechanism inspired the so-called action–observation therapy (AOT), which has been shown to be a valuable rehabilitative tool in several clinical contexts [61]. In this view, the present results suggest that this mechanism might extend rehabilitative opportunities outside the clinical context, toward a more socio-ecological environment, which is represented by the daily activities jointly performed with the patient’s partner. The awareness of the impact of the partner’s motor activity on the patient’s sensorimotor system should lead to a new conceptualization of the facilitatory role of the healthy partner in the health of patients with pathologies that affect the control of voluntary movements.

## Figures and Tables

**Table 1:** *p*-values of the post-hoc multiple comparisons of CC indices. The significance value is 0.05, corrected with Bonferroni to 0.0024 (N = 21). Significantly different comparisons in bold.

*p*_Value	CC_self24_H	CC_self24_P	CC_between_HH	CC_between_PP	CC_self12_H	CC_self12_P
CC_couple_PH	0.5680	0.0429	**0.0000**	**0.0000**	**0.0000**	**0.0000**
CC_self24_H		0.0950	**0.0001**	**0.0000**	**0.0000**	**0.0000**
CC_self24_P			0.3313	**0.0004**	**0.0000**	**0.0000**
CC_between_HH				**0.0000**	**0.0000**	**0.0000**
CC_between_PP					**0.0000**	**0.0000**
CC_self12_H						0.0581

## Data Availability

Source data are available to researchers upon request to the authors.
